# Genomic Comparison of Translocating and Non-Translocating *Escherichia coli*

**DOI:** 10.1371/journal.pone.0137131

**Published:** 2015-08-28

**Authors:** Nathan L. Bachmann, Mohammad Katouli, Adam Polkinghorne

**Affiliations:** Genecology Research Centre, Faculty of Science, Health, Education and Engineering, University of the Sunshine Coast, Sippy Downs, Queensland, 4558, Australia; Centre National de la Recherche Scientifique, Aix-Marseille Université, FRANCE

## Abstract

Translocation of *E*. *coli* across the gut epithelium can result in fatal sepsis in post-surgical patients. *In vitro* and *in vivo* experiments have identified the existence of a novel pathotype of translocating *E*. *coli* (TEC) that employs an unknown mechanism for translocating across epithelial cells to the mesenteric lymph nodes and the blood stream in both humans and animal models. In this study the genomes of four TEC strains isolated from the mesenteric lymph nodes of a fatal case of hospitalised patient (HMLN-1), blood of pigs after experimental shock (PC-1) and after non-lethal haemorrhage in rats (KIC-1 and KIC-2) were sequenced in order to identify the genes associated with their adhesion and/or translocation. To facilitate the comparison, the genomes of a non-adhering, non-translocating *E*. *coli* (46–4) and adhering but non-translocating *E*. *coli* (73–89) were also sequenced and compared. Whole genome comparison revealed that three (HMLN-1, PC-1 and KIC-2) of the four TEC strains carried a genomic island that encodes a Type 6 Secretion System that may contribute to adhesion of the bacteria to gut epithelial cells. The human TEC strain HMLN-1 also carried the invasion *ibe*A gene, which was absent in the animal TEC strains and is likely to be associated with host-specific translocation. Phylogenetic analysis revealed that the four TEC strains were distributed amongst three distinct *E*. *coli* phylogroups, which was supported by the presence of phylogroup specific fimbriae gene clusters. The genomic comparison has identified potential genes that can be targeted with knock-out experiments to further characterise the mechanisms of *E*. *coli* translocation.

## Introduction

Bacterial translocation (BT) is one of the main causes of sepsis in hospitalised and immunocompromised patients and is defined as the passage of viable bacteria (and their products) through the gut epithelium to mesenteric lymph nodes (MLN) and further to the blood and sterile organs [1]. Although *E*. *coli* forms a small population of the gut microbial community, it is one of the most commonly isolated species from surgical patients with septicaemia accounting, in some cases, for up to 37.5% mortality rate [2]. Previous studies have demonstrated that the process of BT occurs independently of the *E*. *coli* gut population size, and that adherence and the subsequent translocation is a selective event [3, 4]. In other words, only a few of the many *E*. *coli* strains found in the gut are capable of adhering to the gut epithelium, and even fewer strains are capable of translocating to extra-intestinal sites. These results suggest that BT is a process dictated to a great extent by the ability of the bacteria to translocate.

None the less, this selective process has been found to occur in different animal hosts, and has led to isolation of *E*. *coli* strains capable of translocating with higher efficiency than other resident *E*. *coli* strains in these particular hosts [4–7]. These include a strain isolated from a case of fatal pancreatitis where the bacterium was found in blood, MLN and peritoneal cavity of the deceased person (HMLN-1) [7], an *E*. *coli* strain isolated from pigs subjected to experimental shock (PC-1)[8], and two strains of *E*. *coli* isolated from rat subjected to starvation with or without haemorrhage (KIC-1 and KIC-2) [5].

Animal studies have shown that the efficiency of translocation is dependent on host specificity with the human and pig translocating *E*. *coli* (TEC) strains having a higher rate of translocation in the pig model than the rat TEC strains [6]. The high translocation rate of the human TEC strain in pigs is not surprising since the physiology of the pig intestine and its microbial community is similar to humans [9, 10].

In order to elucidate the pathogenesis of TEC strains, Ramos et al, (2011) investigated the virulence characteristics and the interleukin-8 response to infection by the above TEC strains [11]. They also tested for the presence of 47 virulence genes associated with intestinal and extra-intestinal *E*. *coli* and found that, among TEC strains, the virulence gene coding for Group III poly sialic capsule synthesis was carried by HMLN-1 and PC-1 only and the enteroaggregative stable toxin-1 (EAST1) gene was carried by KIC-2 only. However, it was found that TEC strains elicited significantly higher IL-8 response in both gastrointestinal cell lines (i.e. Caco-2 and HT-29 cells) and monocyte THP-1 cells than non-TEC strains [11]. These *in vitro* experiments also revealed that most TEC strains (except KIC-1) could adhere with greater efficiency to gut epithelial cell lines than non-TEC strains [11]. The KIC-1 strain adhered to these cells at the same level as non-TEC strains, suggesting that the degree of adhesion is not solely associated with translocation. Based on these results, we postulated that TEC strains might harbour certain properties which are probably not seen in other pathotypes of *E*. *coli* and that they may have unique virulence genes contributing to their translocation.

This pilot study aimed to employ a genomic approach to identify genes and/or genetic mechanisms that could potentially contribute to adhesion and translocation of TEC strains. Genomes of the above TEC and non-TEC strains were sequenced and compared and, here, we report all genetic differences identified between these two groups of *E*. *coli* and discuss their possible role in the translocation ability of these bacteria.

## Materials and Methods

### Bacterial strains and growth condition

Four translocating and two non-translocating *E*. *coli* strains were included in this study. Table 1 shows details of their sources of isolation and other characteristics of each strains. All bacterial strains were grown in Luria-Bertani (LB) broth, to log phase (3–4 h), at 37°C with agitation (100 strokes per min). The cultures were centrifuged and resuspended in phosphate-buffered saline (PBS). Total genomic DNA of the strains was extracted using DNeasy blood and tissue kit (QIAGEN, Australia) as per manufacturer’s instructions. DNA quality from the strains was visually assessed using gel electrophoresis using a 1% agarose (Amresco, Astral Scientific, Australia) gel in 0.6 x Tris/Borate/EDTA (TBE) buffer, pre-stained with ethidium bromide, run at 130 V for 60 min. Bands were visualized under ultraviolet (UV). DNA was then submitted for sequencing on ice.

**Table 1:** Summary of *Escherichia coli* genomes sequenced in this study.

Strain	Source of isolate (reference no.)	No. of Contigs	Genes	Plasmids	Accession Number
Translocating strains (TEC)					
HMLN-1	Isolated from MLNs, blood and peritoneal fluid of a patient with fatal haemorrhagic pancreatitis [7]	59	4678	1	LFKC00000000
PC-1	Isolated from MLNs and blood of pigs subjected to ischemia/ reperfusion or peritonitis [8]	125	4725	1	LFKB00000000
KIC-1	Isolated from MLNs of rats subjected to 24 or 48 h of starvation with and without haemorrhage [5]	70	4590	1	LFKA00000000
KIC-2	Isolated from MLNs of rats subjected to 24 or 48 h of starvation with and without haemorrhage [5].	175	4816	0	LFJZ00000000
Non-translocating strains (controls)					
73–89	Isolated from the caecal epithelium of rats subjected to 48 h of starvation with haemorrhage [11]	124	5110	1	LFJY00000000
46–4	Isolated from the caecal contents of rats subjected to 48 h starvation with haemorrhage [11]	66	4595	1	LFJX00000000

### Genome sequencing and phylogenetics

Genomic DNA of all six strains was sequenced at the Australian Genome Research Facility (AGRF) using an Illumina MiSeq to produce paired-end 101-bp reads. Read quality was checked with FASTQC and Spades 3.0.0 was used to assemble the *E*. *coli* genomes with k-mer values of 15, 21, 33, 51 and 71 [12] The evolutionary relationship of the *E*. *coli* strains sequenced in this study compared to 29 complete *E*. *coli* and one *E*. *fergusonnii* genome was predicted by phylogenetic analysis using concatenated nucleotide sequences of seven housekeeping genes (*adk*, *fumC*, *gyrB*, *icd*, *mdh*, *purA* and *recA*), as previously described [13]. Sequences were aligned in Muscle v3.8.31 with default settings. The Neighbour-Joining method of MEGA5 was used to infer the evolutionary history, with distances computed by the Jukes-Cantor method. The phylogenetic tree was rooted using *E*. *fergusonnii* as an out-group.

### Genome comparative analysis

The Rapid Annotation using Subsystems Technology (RAST) server provided annotation for the six *E*. *coli* genomes. Manual curation was also performed to ensure the accuracy of the annotation with particular attention to regions of difference, prophage regions and genomic islands using Artemis [14]. BLAST ring image generator (BRIG) [15], Easyfig [16] and Artemis Comparison Tool (ACT) [17] were used to visualise the comparison of the six *E*. *coli* genomes sequenced in this study and 41 previously completed *E*. *coli* genomes (S1 Table). The presence/absence of chaperone-usher (CU) fimbriae gene clusters were determined with BLAST using 38 CU fimbrial operons defined in Wurpel *et al* [18] and visualized with BRIG.

## Results

### Draft genomes of TEC

The draft genomes of four previously described efficiently translocating *E*. *coli* strains isolated from humans (HMLN-1), pigs (PC-1) and rats (KIC-1 and KIC-2) were sequenced. The four TEC strains were selected based on their ability to adhere and translocate across human gut epithelial Caco-2 and HT-29 cells [11]. The draft genomes were assembled into 59 contigs for strain HMLN-1, 125 contigs for PC-1, 70 contigs for KIC-1 and finally 175 contigs for the strain KIC-2 (Table 1). In addition, draft genomes were sequenced from two non-translocating *E*. *coli* strains (46–4 and 73–89) isolated from rats. The non-TEC strains were used as controls for testing TEC strain phenotypes [11]. The draft genomes of *E*. *coli* strains 46–4 and 73–89 assembled into 66 contigs and 124 contigs, respectively. Three of the TEC strains (HMLN-1, PC-1 and KIC-1) and both non-TEC strains genomes carried a single plasmid ranging from 53 kbp to 101 kbp that encodes a conjunctive transfer system and several hypothetical proteins. No plasmids were found in the genome of the strain KIC-2.

### Relationship between TEC and other *E*. *coli* pathotypes

**Fig 1:**
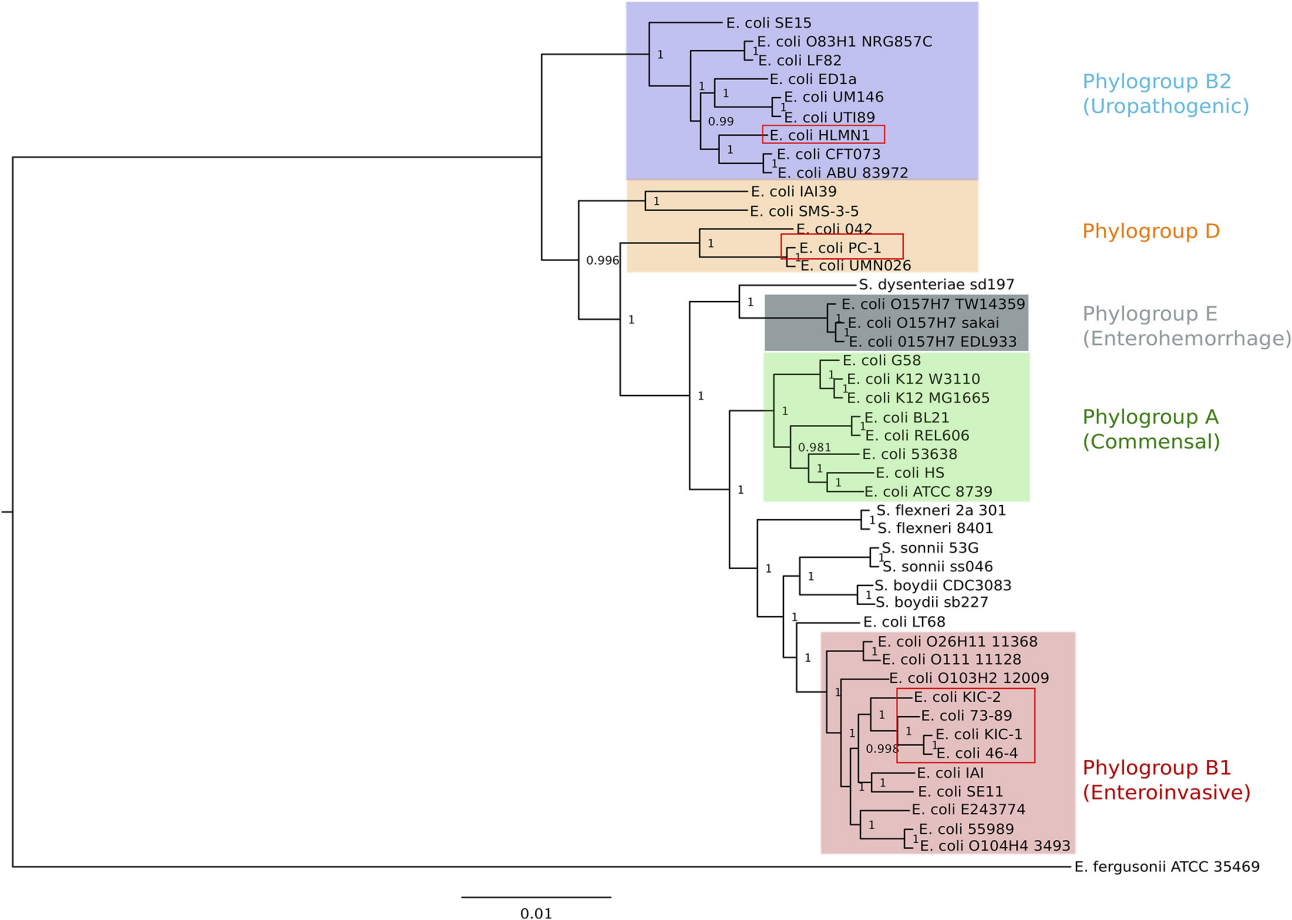
Evolutionary context of translocating *E*. *coli*. The phylogeny of *E*. *coli* strains inferred using the Neighbour Joining method on the concatenated nucleotide sequence of seven housekeeping genes (~9 kb) with *E*. *fergusonii* ATCC 35469 as an out-group. Coloured boxes show the phylogroups (A, B1, B2, D and E). *E*. *coli* strains sequenced in this study are marked with red boxes.

To determine the relationship between the TEC strains sequenced in this study and other *E*. *coli* strains, a neighbour-joining phylogenetic tree was constructed using the concatenated nucleotide sequence of seven housekeeping genes with a total length of 9016 bp. The four TEC strains were separated into three distinct phylogenetic groups (Fig 1). The *E*. *coli* population is often divided into five phylogenetic groups (A, B1, B2, D and E) based on differences in their phylogenetic relationships [19]. The human and the pig strains belong to the phylogenetic groups B2 and D, respectively. These two phylogroups usually contain extraintestinal pathogenic *E*. *coli* (ExPEC) strains [20, 21]. On the other hand, the rat TEC strains KIC-1 and KIC-2 and the two non-TEC strains 46–4 and 73–89 belonged to the B1 phylogroup, which together with phylogenetic group A, are considered as commensals [22, 23] or intestinal pathogenic strains.

### Genomic features of translocating *E*. *coli* strains

**Fig 2:**
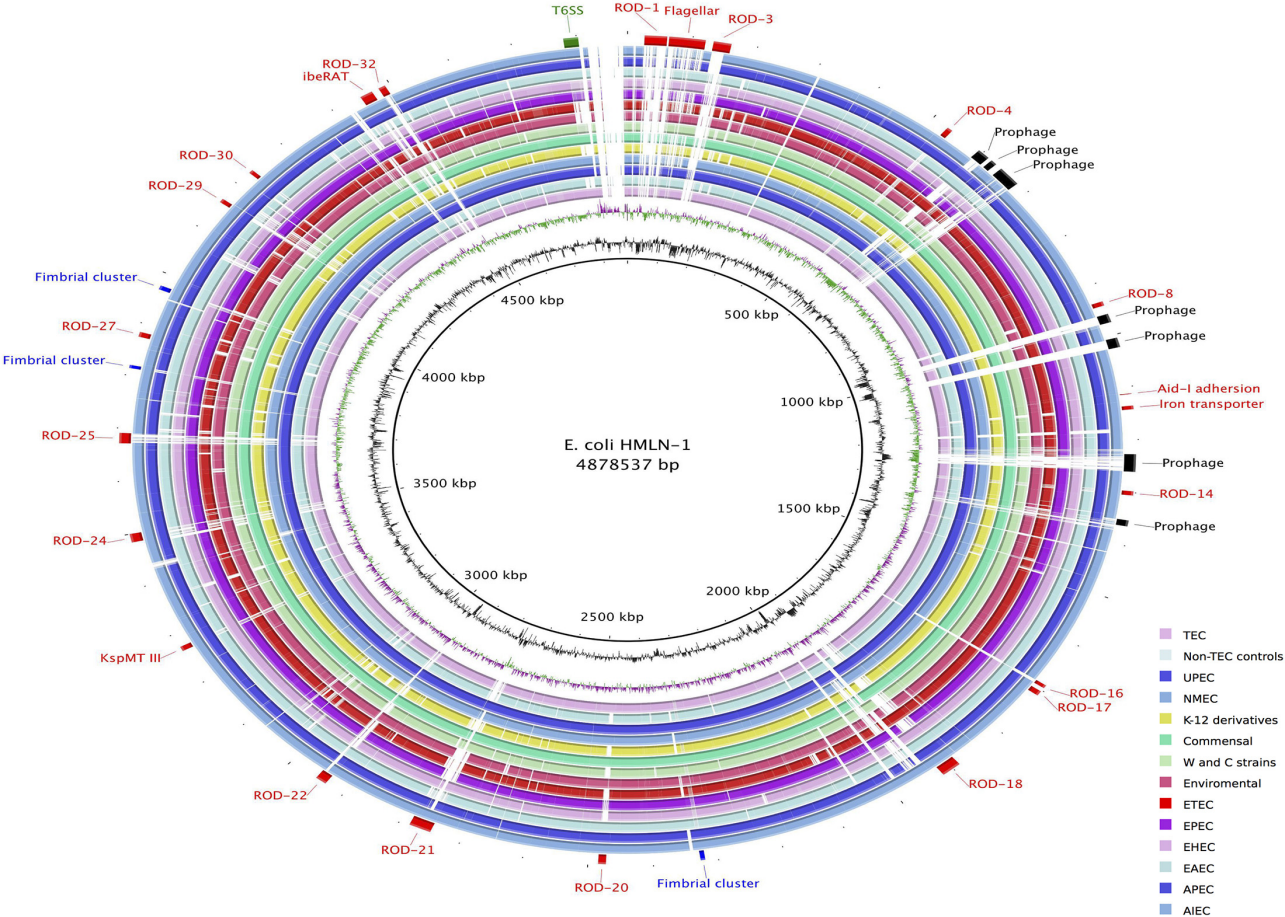
Unfiltered BLASTn comparison of the *E*. *coli* HMLN-1 compared against selected *E*. *coli* genomes coloured according to pathotype or strain group (collapsed into individual rings). The innermost rings represent the GC content (black) and GC skew (purple/green) of the *E*. *coli* HLMN-1 genome. The subsequent coloured rings show the results of BLAST searches of *E*. *coli* genomes against HLMN-1. Inner to outer coloured circles are as follows: TEC strains PC-1, KIC1 and KIC2; Non-TEC strains 73–89 and 46–4; UPEC strains UTI89, 536, ABU83972, CFT073, IAI39 and UMN026; NMEC strains IHE3034 and S88; K-12 derivative strains W3110, MG1655 and DH10B; commensal strains BL21, REL606, SE11, IAI1, HS, ED1a and SE15; clone W and ATCC8739 (clone C) strains; Environmental SMS-3-5 strain; Enterotoxigenic *E*. *coli* (ETEC); Enteropathogenic *E*. *coli* (EPEC); Enterohemorrhagic *E*. *coli* (EHEC); Enteroaggregative *E*. *coli* (EAEC); Avian pathogenic *E*. *coli* (APEC) and adherent-invasive *E*. *coli* (AIEC). Colour intensity is proportional to BLASTn identity with bold regions having high nucleotide identity and light regions having low nucleotide identity. *E*. *coli* HLMN-1 genomic features are annotated around the outmost ring: prophages regions in black, fimbria clusters in blue, Type VI Secretion System in green and other features and region of differences (RODs) in red.

**Fig 3:**
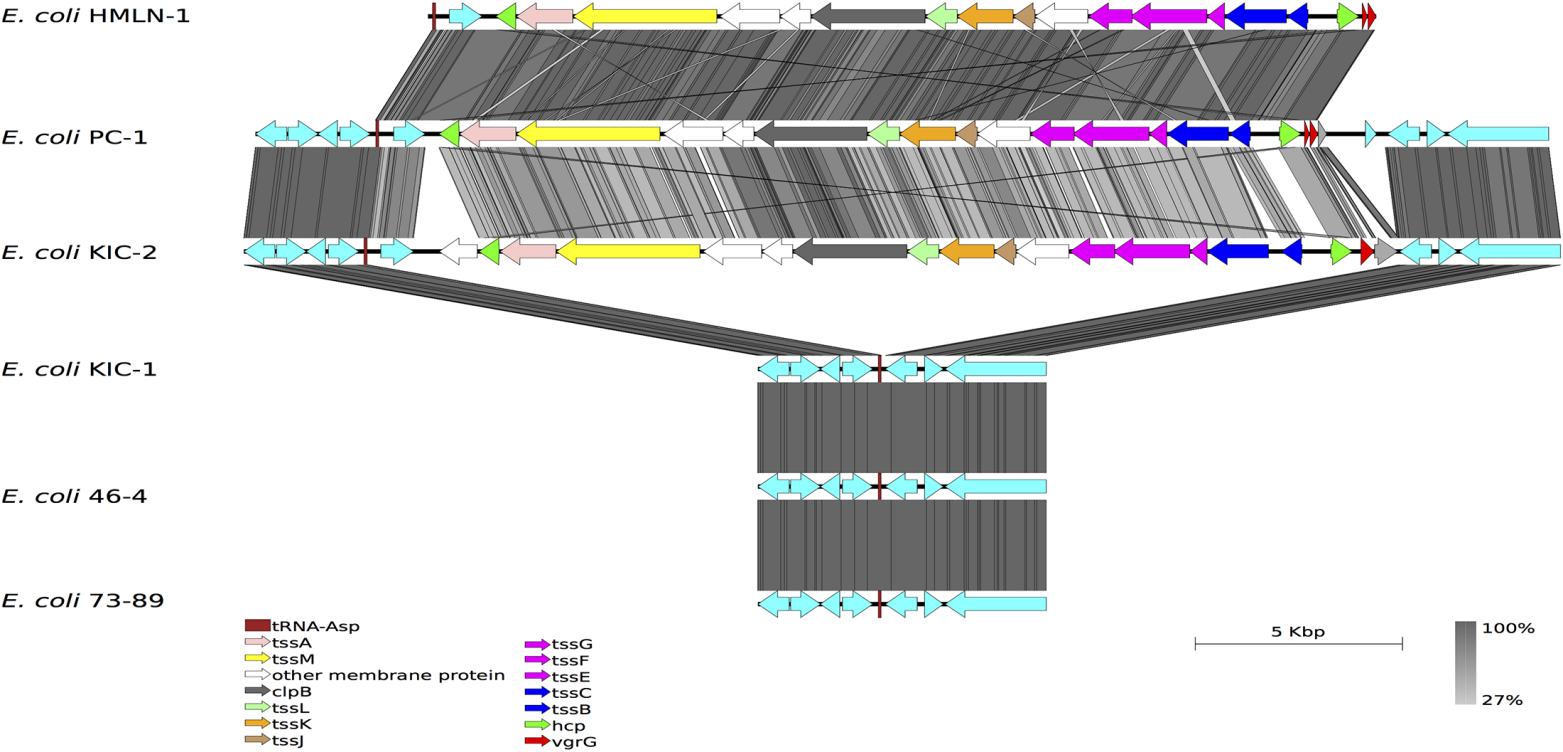
Visual comparison of the TEC Type VI Secretion System. tBLASTx comparison of the Type VI Secretion System (T6SS) genomic island and flanking regions in *E*. *coli* HLMN-1, *E*. *coli* PC-1, *E*. *coli* KIC-1, *E*. *coli* KIC-2, *E*. *coli* 46–4 and *E*. *coli* 73–89. Grey vertical blocks indicated regions of conserved nucleotide sequence determined using tBLASTx and the arrows represent genes and are coloured.

The genomes of the four TEC strains were compared to the two non-TEC genomes to determine the genetic differences that may be linked to adhesion and translocation across epithelial cells. The comparisons were also extended to include 41 publically available *E*. *coli* genomes representing all the major pathotypes. The comparison revealed that the genomic backbone of *E*. *coli* HMLN-1 was most similar to the genomes of Adherent-Invasive *E*. *coli* (AIEC) strains LF82, NRG857C and UM146. In *E*. *coli* HMLN-1, we have defined 29 regions of differences (RODs) and five putative prophage regions that are sporadically distributed amongst the other 46 *E*. *coli* genomes (Table 2 and Fig 2). The five putative prophage regions, of which three are unique to HMLN-1, could be distinguished from the *E*. *coli* genomic backbone by the presence of phage structural/replication genes and skews in the average GC-content. Amongst the RODs, the most significant was the presence of the genomic island GI-argU, which encodes a Type VI Secretion System (T6SS). GI-argU is a 22.4 kbp region inserted next to the tRNA^argU^ and was present in three of the TEC strains; HMLN-1, PC-2 and KIC-2 but absent in KIC-1 and both of the non-TEC control strains, *E*. *coli* 46–4 and 73–89 (Fig 3) Genes encoding for a T6SS are commonly found in most other *E*. *coli* pathotypes but they are absent in the K-12 derivatives, environmental and enterotoxigenic *E*. *coli* strains (Fig 2). The 18 genes located within the GI-argU region in *E*. *coli* HMLN-1, PC-1 and KIC-2 encode all the structural proteins of the T6SS and the two T6SS-associated secreted proteins; hemolysin-coregulated protein (Hcp) and valine-glycine repeat G (VgrG) [24].

**Table 2:** Major regions of difference in *E*. *coli* HMLN-1.

Identifier	Contig	Start	End	Size (bp)	Insertion site	No. of genes	GC Content (%)	Description	Notable virulence factor
ROD-1	3	2182	36168	33986	-	40	42	Hypothetical proteins	
ROD-2	3	40411	54202	13791	-	14	45	Flagellar	
ROD-3	4	54244	81785	27541	tRNA-Thr-CGT	26	39	Sugar transportor	
ROD-4	7	8799	17778	8979	-	9	52	Glycoside degradation	
ROD-5	8	1	20003	20002	tRNA-Lys-TTT	22	46	Prophage	
ROD-6	8	26182	36669	10487	-	10	45	Prophage	
ROD-7	9	1	35407	35406	-	51	46	Prophage	
ROD-8	9	292659	300796	8137	-	6	43	Curli biogenesis	
ROD-9	9	317516	333578	16062	-	16	36	Prophage	
ROD-10	10	1	17530	17529	tRNA-Lys-TTT	20	37	Prophage	
ROD-11	10	106173	107692	1519	-	3	42	Aid-I adhersion	
ROD-12	10	129437	135863	6426	-	6	52	Iron transporter	
ROD-13	11	6885	39719	32834	-	35	54	Prophage	
ROD-14	11	76907	84399	7492	-	5	51	RND multidrug effux pump	
ROD-15	11	131644	140713	9069	-	12	46	Prophage	
ROD-16	17	240004	245998	5994	-	3	42	sel1 protein	
ROD-17	17	253605	262317	8712	-	8	46	thiamine biosynthesis	
ROD-18	18	1	30512	30511	tRNA-Asn-GTT	12	58	ABC transporter	
ROD-19	18	433292	440290	6998	-	7	51	Fimbrial cluster	
ROD-20	19	90511	102325	11814	-	4	50	Virulence related	RatA, SinI
ROD-21	22	1	34254	34253	tRNA-Met-CAT	25	52	uncharacterized Type VI-like secretion system	
ROD-22	22	184315	202088	17773	tRNA-Phe-GAA	12	42	Capsular polysaccharide export system	KspMT III
ROD-23	22	516756	525252	8496	-	8	46	Ribose ABC transporter system	
ROD-24	26	25349	41193	15844	-	16	48	metabolic enzymes	
ROD-25	28	99422	118783	19361	tRNA-SeC(p)-TCA	22	43	Hypothetical proteins	
ROD-26	28	241596	247361	5765	-	5	45	fimbrial cluster	
ROD-27	29	11045	19997	8952	-	9	50	Transporters	
ROD-28	29	104873	112534	7661	-	7	39	fimbrial cluster	
ROD-29	31	50454	57616	7162	-	5	51	Hypothetical proteins	
ROD-30	31	121132	127253	6121	-	6	49	D-allose ABC transporter	
ROD-31	31	342824	363000	20176	YjiD	14	46	phosphotransferase and ibeRAT operon	IbeA
ROD-32	31	374777	386028	11251	-	7	46	Type I restriction modification system	
ROD-33	32	1	22898	22897	-	19	53	Type VI secretion system	

A significant region in *E*. *coli* HMLN-1 was ROD-31, which was 20.3 kbp in length and contained the virulence *ibeRAT* operon. ROD-31 was present in *E*. *coli* HMLN-1 but absent in the other TEC strains and both of the non-TEC strains and it can also be found in extraintestinal pathogenic *E*. *coli* (ExPEC). The *ibeRAT* is a three-gene operon encoding a regulatory protein (IbeR) and two invasion proteins (IbeA and IbeT). The role for IbeA in pathogenesis has been reported for *E*. *coli* such as avian pathogenic *E*. *coli* and adherent-invasive *E*. *coli* and is linked with invasion of the intestinal epithelium and survival in macrophages [25]. The IbeT protein has been shown to affect adhesion and invasion of brain endothelial cells [26]. The *ibeR* gene encodes an RpoS-like regulator that is predicted to regulate the *ibeA* and *ibeT* genes. Another important feature of *E*. *coli* HMLN-1 genome was the presence of a 17 kbp genomic island (ROD-22), which was inserted next to tRNA^Phe^ and contained the components necessary for the expression of Group III capsular polysaccharide. ROD-22 includes the genes, *kpsM* and *kpsT*, whose products are critical for the export of the polysaccharide capsule [27].

The *E*. *coli* PC-1 genome contained 12 RODs that were not found in the non-TEC strains and three of which encode proteins that are homologous with proteins that have adhesion and invasion functions (S2 Table). In the genome of the rat TEC strain *E*. *coli* KIC-2 there were 14 RODs, which included six prophages (S3 Table) while the *E*. *coli* KIC-1 had only three RODs (S4 Table). The extra gene content of KIC-1 and KIC-2 has no suggested roles in translocation. However, the KIC-2 genome did carry additional mechanisms potentially associated with adhesion (T6SS and Aid-I like adhesion protein) while KIC-1 did not, which is supported by the higher adhesion rate seen in *in vitro* experiments with KIC-2 [11].

### Fimbria clusters of TEC

**Fig 4:**
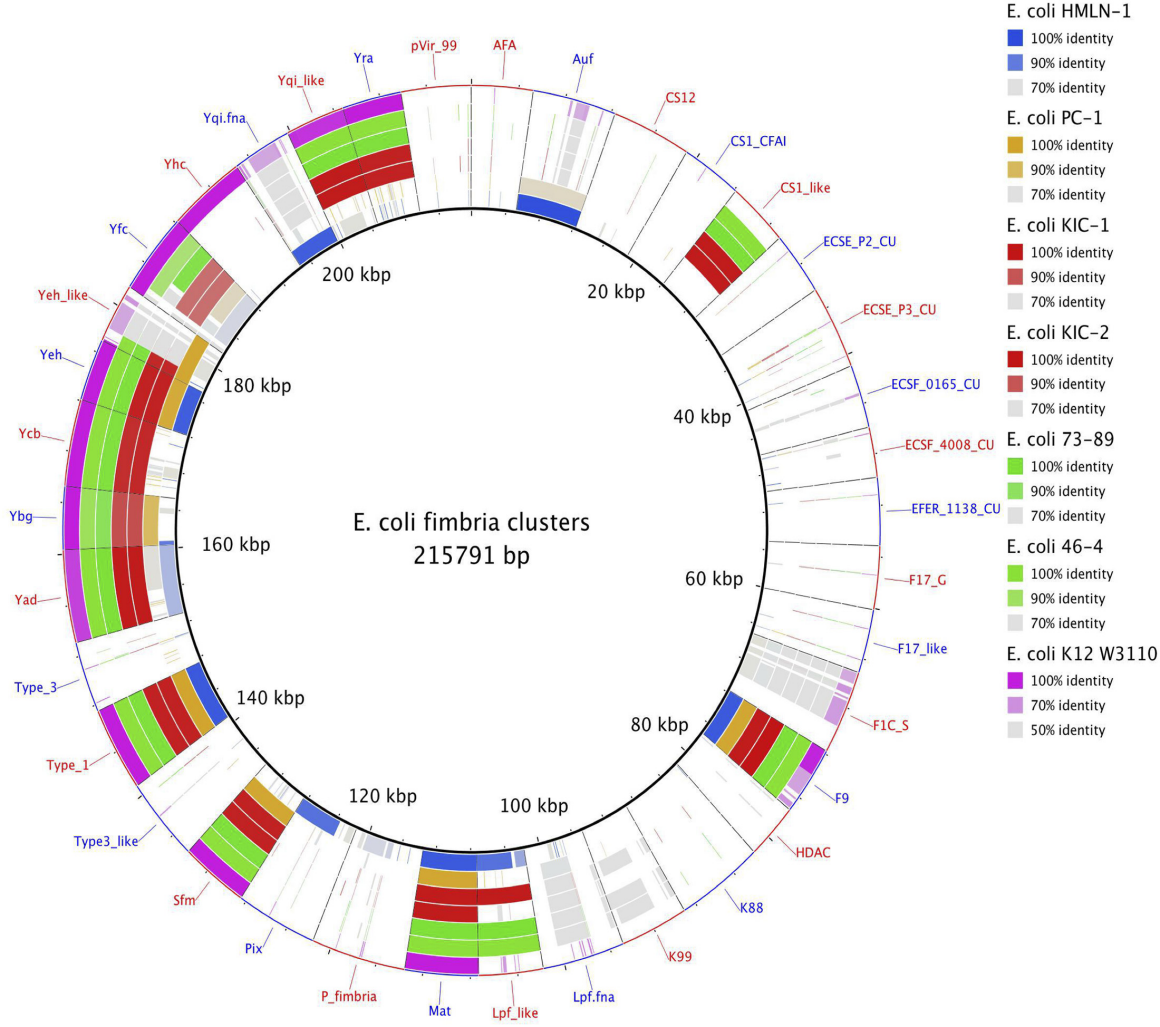
Distribution of chaperone-usher (CU) fimbrial gene clusters in translocating *E*. *coli*, control *E*. *coli* strains sequenced in this study and *E*. *coli* K-12 W3110. The inner ring represents the concatenated nucleotide sequences of 38 fimbrial operons. Subsequent rings indicate the presence of the CU fimbrial gene clusters in each genome. Each segment is labelled according to the name of the CU fimbrial families.

The presence and distribution of chaperone-usher (CU) fimbrial gene clusters were investigated to see if there was a link between fimbriae and the observed phenotypes of the TEC strains. Fig 4 shows the distribution of 38 CU fimbrial operons in the four TEC strains and the two non-TEC strains as well as a reference strains, *E*. *coli* K-12 W3110. The distribution of CU fimbrial operons in the TEC strains was consistent with the phylogroups that each of the strains belonged too. All the TEC and non-TEC strains possessed the core-associated *E*. *coli* CU fimbrial operons including the Type 1, Yad, Yeh, Yfc, Mat, F9 and Ybg fimbriae. The sfm fimbriae was present in all TEC and non-TEC genomes sequenced in this study expected for *E*. *coli* HMLN-1, however, the sfm fimbriae is usually absent in *E*. *coli* strains from the B2 phylogroup [18]. In addition to the core CU fimbrial operons, the TEC strains KIC-1 and KIC-2 as well as the non-TEC strains 46–4 and 73–89 also carried clade-specific CU fimbriae commonly found in strains that are part of the B1 phylogroup such as CS1-like, Yra, Yqi-like and Ycb [18]. *E*. *coli* HMLN-1 carried three B2-clade specific CU fimbrial gene clusters (Pix, Yqi and P-fimbriae) that were not present in the other genome sequences. The intact Auf fimbrial operon that is commonly found in both B2 and D phylogroup strains was present in both the HMLN-1 and PC-1 genomes.

## Discussion

Translocating *E*. *coli* is a unique pathogroup that was identified by their ability to translocate across gut epithelial cells in animals and humans [6]. The human TEC strain tested in this study was associated with a case of fatal haemorrhagic pancreatitis suggesting that TEC strains are a public health concern [7]. However, it is currently unknown what genes are involved in translocation since the most common *E*. *coli* virulence genes are absent in TEC strains [11]. Therefore, this study employed whole genome sequencing to compare TEC and non-TEC strains to identify the genes involved in the unique phenotypes exhibited by this pathogroup. The *E*. *coli* strains selected for sequencing were TEC strains that were previously tested for adhesion and translocation in animal studies [4] and in *in vitro* experiments [11]. In addition, non-TEC strains that were used as a control in the *in vitro* experiments were sequenced so that the genomes can be compared to identify regions of differences that may be associated with the translocation phenotype.

The phylogenetic relationship of the TEC strains have been previously explored using an unweighted pair group method with arithmetic averages (UPGMA) approach based on the similarity between the biochemical phenotypes of these strains [11]. However, using the genome sequences a more accurate phylogenetic relationship was determined for the TEC strains. The neighbour joining tree based on seven housekeeping genes employed in this study, showed that the TEC strains were distributed amongst three distinct *E*. *coli* phylogroups. The dissemination of the TEC strains in the *E*. *coli* phylogenetic tree are independent of disease phenotype, similar to what has been observed with AIEC strains [28]. Most of the TEC strains were part of the same phylogroup that was predicted with the UPGMA approach. However, in the neighbour joining tree, *E*. *coli* HMLN-1 was part of phylogroup B2 instead of phylogroup D as originally predicted [11]. *E*. *coli* strains belonging to this phylogenetic group have been associated with pyelonephritis and meningitis [19, 29]. The closely related TEC strain to HMLN-1 was the PC-1 strain, which was originally isolated from the blood of pigs subjected to pancreatitis and ischemic reperfusion. These two strains have previously been shown to have identical biochemical phenotypes and serotypes [11] however, the PC-1 strains belonged to phylogenetic group D which is also found among UPEC strain. The KIC-1 and KIC-2 strains together with the non-TEC strains isolated from rats belonged to phylogroup B1, which are previously regarded as commensal gut strains. Ramos *et al*, [11] showed that these rat-origin TEC strains were not as efficient at translocating in a cell line representing human gut epithelium as HMLN-1 and PC-1, although they translocated efficiently in rats [4].

A genomic island encoding a T6SS was found in the three TEC strains (HMLN-1, PC-1 and KIC-2) that can efficiently adhere to epithelial cells [11]. The T6SS however, was absent in *E*. *coli* KIC-1 and the two non-TEC strains, which adhered to epithelial cells to a lesser degree than the other strains. The T6SS is commonly found in pathogenic *E*. *coli* strains isolated from patients with inflammatory bowel disease [30]. The T6SS has also been identified in commensal *E*. *coli*, including *E*. *coli* strain W [31]. The presence of similar T6SS in both pathogenic and commensal *E*. *coli* suggests that it has a role in pathogenesis and/or intra-bacterial competiveness [32]. The T6SS consists of 14 genes that encode for the core structural apparatuses. Studies of other bacterial pathogens have revealed that the T6SS is involved in adhesion to host cells [33, 34]. Therefore, the presence of the T6SS in TEC strains with a high adhesion rate and its absent in the TEC strain KIC-1 suggests that the T6SS plays a role in adhesion for TEC strains rather than translocation. Another unique finding of this comparative study was the presence of the *ibe*RAT operon in *E*. *coli* HMLN-1 that was missing from other TEC strains. The *ibe*A and *ibe*T genes encodes for invasive proteins [26] while the *ibe*R gene encodes a regulator [35]. Experimental work on *ibeA* in *E*. *coli* has shown that the encoded protein contributes to invasion by improving bacterial survival in macrophages by functioning as an oxidoreductase, which provides resistance to reactive oxygen species [25, 36]. The presence of the *ibe*A gene likely contributes to the pathogenic potential of *E*. *coli* HMLN-1 and may assist with translocation. However, the absence of the *ibe*A gene in the other TEC strains suggest that there are other mechanisms associated with translocation, which may include other unknown invasion genes located on the chromosome or plasmids. It is also possible that *ibe*A gene is involved in a host-specific adhesion mechanism, a view which is supported by the study of Katouli *et al* [4] who showed that translocation of these four TEC strains was host-specific.

Fimbriae are long proteinaceous organelles that extend from the surface of many bacteria and mediate diverse functions, including adherence and biofilm formation [37]. In Gram-negative bacteria, one of the ways those fimbriae are assembled and transported from the cytoplasm to the cell surface is via the chaperone-usher pathway [38]. The fimbriae structure genes are clustered together with the chaperone and usher genes. A previous study has identified 38 distinct CU fimbrial operons in *E*. *coli* based on an usher protein phylogeny [18]. The presence of clade specific CU fimbriae operons in TEC strains was consistent with the phylogroups that each strain belonged too. In addition, the presence of the Ygi and Pix CU fimbrial operons in *E*. *coli* HMLN-1 further support that this strain belongs to the B2 phylogroup, since the Ygi and Pix fimbriae are only found in strains from the B2 phylogroup [18]. These observations therefore suggest that the variation in the CU operons is not linked to TEC strain adhesion and translocation phenotypes.

In conclusion, the genomic comparison strongly suggests that the T6SS is involved in adhesion of the TEC strains to epithelial cells rather than translocation. The invasion gene *ibe*A in *E*. *coli* HMLN-1 may also contributed to translocation and disease outcome of this particular TEC strain. It is likely that translocation is mediated by an assortment of different invasion proteins that may vary between strains from different hosts. A future direction for the investigation of TEC will be to “knock-out” the T6SS ATPase gene and the *ibe*A gene in the *E*. *coli* HMLN-1 to determine the effect they may have on adhesion and translocation.

## Supporting Information

S1 Table List of published *E*. *coli* genomes used in the comparative analysis. (XLSX)

S2 Table Major regions of difference in *E*. *coli* PC-1. (XLSX)

S3 Table Major regions of difference in *E*. *coli* KIC-2. (XLSX)

S4 Table Major regions of difference in *E*. *coli* KIC-1. (XLSX)
